# Epigenome-wide association study and epigenetic age acceleration associated with cigarette smoking among Costa Rican adults

**DOI:** 10.1038/s41598-022-08160-w

**Published:** 2022-03-11

**Authors:** Andres Cardenas, Simone Ecker, Raj P. Fadadu, Karen Huen, Allan Orozco, Lisa M. McEwen, Hannah-Ruth Engelbrecht, Nicole Gladish, Michael S. Kobor, Luis Rosero-Bixby, William H. Dow, David H. Rehkopf

**Affiliations:** 1 Division of Environmental Health Sciences, School of Public Health and Center for Computational Biology, University of California, Berkeley, 2121 Berkeley Way, #5121, Berkeley, CA 94720 USA; 2 UCL Cancer Institute, University College London, London, UK; 3 School of Medicine, University of California San Francisco, San Francisco, CA USA; 4 School of Health Technology, Faculty of Medicine, University of Costa Rica (UCR), San José, San Pedro, Costa Rica; 5 Faculty of Human and Social Development, School of Health Information Science, University of Victoria, Victoria, BC Canada; 6 Department of Medical Genetics, Centre for Molecular Medicine and Therapeutics, and BC Children’s Hospital Research Institute, University of British Columbia, Vancouver, BC Canada; 7 Centro Centroamericano de Población (CCP), Universidad de Costa Rica, San José, Costa Rica; 8 Division of Health Policy and Management, School of Public Health, University of California, Berkeley, Berkeley, CA USA; 9 Department of Epidemiology and Population Health and Department of Medicine, School of Medicine, Stanford University, Palo Alto, CA USA

**Keywords:** DNA methylation, Epidemiology

## Abstract

Smoking-associated DNA methylation (DNAm) signatures are reproducible among studies of mostly European descent, with mixed evidence if smoking accelerates epigenetic aging and its relationship to longevity. We evaluated smoking-associated DNAm signatures in the Costa Rican Study on Longevity and Healthy Aging (CRELES), including participants from the high longevity region of Nicoya. We measured genome-wide DNAm in leukocytes, tested Epigenetic Age Acceleration (EAA) from five clocks and estimates of telomere length (DNAmTL), and examined effect modification by the high longevity region. 489 participants had a mean (SD) age of 79.4 (10.8) years, and 18% were from Nicoya. Overall, 7.6% reported currently smoking, 35% were former smokers, and 57.4% never smoked. 46 CpGs and five regions (e.g.* AHRR*, *SCARNA6*/*SNORD39*, *SNORA20*, and *F2RL3*) were differentially methylated for current smokers. Former smokers had increased Horvath’s EAA (1.69-years; 95% CI 0.72, 2.67), Hannum’s EAA (0.77-years; 95% CI 0.01, 1.52), GrimAge (2.34-years; 95% CI1.66, 3.02), extrinsic EAA (1.27-years; 95% CI 0.34, 2.21), intrinsic EAA (1.03-years; 95% CI 0.12, 1.94) and shorter DNAmTL (− 0.04-kb; 95% CI − 0.08, − 0.01) relative to non-smokers. There was no evidence of effect modification among residents of Nicoya. Our findings recapitulate previously reported and novel smoking-associated DNAm changes in a Latino cohort.

## Introduction

Cigarette smoke causes adverse health outcomes; however, smoking cigarettes remains a common behavior around the world^1^. The smoke is comprised of a complex chemical mixture of over 7000 compounds, including multiple known human carcinogens^2^. Smoking is a major environmental risk factor for the development of respiratory and cardiovascular illnesses, including chronic obstructive pulmonary disease and coronary heart disease^3,4^. Smoking cigarettes has deleterious effects on multiple organs throughout the body and is both the largest preventable cause of cancer-related deaths^5–7^ and the leading intervenable cause of death in the United States, greater than diet and physical activity patterns combined^8,9^. Biological pathways implicated in the smoking-induced pathogenesis of diseases include inflammation, oxidative stress, cellular apoptosis, extracellular matrix destruction, and impaired cellular signaling^10,11^. Growing evidence suggests that both direct DNA damage and epigenetic mechanisms play major roles in smoking-associated diseases.

A common epigenetic modification is DNA methylation, the addition of a methyl group to a cytosine nucleotide followed by a guanine (CpG), which can regulate gene expression. Multiple epigenome-wide association studies (EWAS) conducted in blood samples have shown that adult cigarette smoking is associated with altered DNA methylation patterns of leukocytes across cohorts of mostly European descent^12–18^. These smoking-associated changes in DNA methylation may contribute to an increased risk for poor health outcomes among smokers. For example, studies have consistently found hypomethylation of the *F2RL3* (coagulation factor II receptor-like 3) and *AHRR* (aryl hydrocarbon receptor repressor) genes in smokers compared to non-smokers, which, in turn, has been associated with reduced lung function and increased mortality^13,19–22^. Most studies and meta-analyses have tested DNA methylation changes with the 450K methylation array (Infinium HumanMethylation450 BeadChip; Illumina), and only few have used the newer and more comprehensive 850K methylation array (Infinium HumanMethylation EPIC BeadChip; Illumina), which may provide further insights^23^. Furthermore, the reproducibility of smoking-associated DNA methylation signatures in non-Caucasian populations is not well established.

Other important biomarkers to elucidate the effects of environmental exposures, specifically on the aging process and age-related diseases, are epigenetic clocks. Epigenetic clocks use DNA methylation levels of age-associated CpG sites to estimate an epigenetic age that can then be compared to chronological age in order to determine age acceleration, a measure of biological aging^24^. Among adults, increased epigenetic age acceleration is associated with factors like an unhealthy diet, lack of exercise, and lifetime stress and has been shown to help evaluate susceptibility to diseases like lung cancer^25–28^. Several epigenetic clocks have been developed in order to include epigenetic markers in specific or multiple tissues and improve predictive performance for specific aging measures, morbidity, or mortality. These include the Hannum blood^29^, Horvath Pan Tissue^24^, Skin-Blood^30^, PhenoAge^31^, and GrimAge^32^ clocks. Additionally, telomere length can be estimated from DNA methylation (DNAmTL), which more closely correlates with chronological age relative to measured telomere length^33^. The GrimAge clock is particularly successful at predicting mortality associated with factors like smoking and obesity^32^. Unlike the other clocks, GrimAge was trained on smoking pack-years and includes chronological age in its input. Cigarette smoking has recently been shown to be associated with increased age acceleration in respiratory tissues, which may be reversed by smoking cessation^34^.

Overall, there is a lack of EWAS data and epigenetic age acceleration studies on smoking conducted with diverse populations, including Latinos, in which three of the top five populations-specific causes of death—cancer, stroke, and heart disease—are all associated with smoking^35^. Studying the epigenetic effects of smoking in a Latino population could contribute to characterizing health disparities this group experiences^36,37^ as well as assess the generalizability of findings from other studies, as epigenetic analyses on the effects of smoking have been found to differ by ethnic groups^14^. In this study, we investigated DNA methylation patterns of current and former smokers compared to non-smokers in people living in Costa Rica in order to address the lack of studies in Latino populations. The study population includes participants from the Nicoya peninsula: a “Blue Zone” characterized by exceptionally high longevity compared to the rest of Costa Rica and the world^38,39^. We also use multiple epigenetic aging biomarkers to understand how smoking may impact biological aging of participants. We hypothesized that most previously identified smoking signatures would be generalizable to this Latino cohort and that study participants from the high longevity region would exhibit epigenomic resiliency to smoking-associated epigenetic changes and epigenetic age acceleration.

## Methods

### Data collection and sample preparation

The study participants were selected from The Costa Rican Study on Longevity and Healthy Aging (CRELES) cohort; the study protocol has been previously described^40–42^. Briefly, CRELES is a prospective longitudinal study of a nationally representative sample of 2827 residents of Costa Rica who were age 60 years and older at baseline in 2004–2006, with a second wave of interviews and data collection in 2006–2008. Information from a CRELES-complementary sample of Nicoyan quasi-centenarians (age 95 and above) were also collected*.* All data, examinations, and specimens were taken in the participants’ homes, and details about sample, field, and laboratory procedures have been previously reported^40,41^. The Ethical Science Committee of the University of Costa Rica granted human subjects approval to CRELES (VI-763-CEC-23-04). All participants granted written informed consent by means of their signature and the study was conducted according to the guidelines laid down in the Declaration of Helsinki.

We randomly selected 512 individual samples from both wave 1 and wave 2 blood samples for DNA methylation (DNAm) analysis. We ascertained smoking behavior by interviews and classified participants as current smokers if they reported smoking > 100 cigarettes in their lifetime as well as currently smoking at the study visit. Former smokers reported smoking > 100 cigarettes in their lifetime but not currently smoking at the study visit, while non-smokers reported not smoking over > 100 cigarettes in their lifetime and currently not smoking. We also investigated ever smoking (> 100 cigarettes in their lifetime) vs. non-smokers for comparability with previous studies.

### DNA methylation measurements

Whole blood samples were collected via venipuncture and processed at the University of Costa Rica, as previously described^43^. Genomic DNA was extracted from 2 mL of frozen whole blood using the phenolchloroform method. DNA was bisulfite converted with the Zymo Research EZ DNA Methylation™ Kit (Irvine, CA, USA). Bisulfite-converted DNA from each sample was randomized across Infinium MethylationEPIC BeadChips as well as sentrix row and run in one batch according to the manufacturer’s protocol (San Diego, CA, USA)^42^.

We processed raw DNA methylation image files using the *R* statistical software (www.r-project.org/) and several Bioconductor packages^44^ including the *minfi* pipeline for quality control^45^. All samples had median methylated and unmethylated log-intensities above a threshold considered to be of good quality (> 10.5). We used functional normalization with 3 principal components capturing > 90% of the variation in the control probes to normalize samples. We chose this normalization method given that participants came from two geographic regions in Costa Rica, and we expected genetic ancestry differences that could influence DNA methylation by region. A total of 512 samples were analyzed, and 12 samples were identified as outliers based on principal component analyses^29^ or by having ≥ 5% of CpGs with non-significant detection (*P* > 1 × 10^–16^). We also removed 3 samples mismatched on recorded sex and 8 technical replicates, leaving a total of 489 samples. Data from the 489 participants from waves 1 (n = 274) and 2 (n = 215) were included after quality control. We removed 59 SNP probes, probes with < 3 beads or with a non-significant detection (*P* > 1 × 10^–16^) in ≥ 1% of samples, removing a total of 22,261 non-reliable probes. We further removed 18,474 probes in XY chromosomes and 25,395 polymorphic probes containing a SNP at the CpG site or single base extension with a minor allele frequency > 1%. We removed 9671 autosomal probes that cross-hybridize to sex chromosomes^46^. A total of 790,058 high quality CpGs were used in statistical analyses. Finally, we corrected for sample plate, row (position within the array), and chip using ComBat^47^. We visualized the density distributions for samples at all processing steps and performed principal components (PC) analyses to examine the associations of methylation differences with technical, biological, and measured traits with global DNA methylation variation. For each CpG site, methylation is reported as the average β-value, corresponding to an interval scaled quantity between zero and one interpreted as the fraction of DNA molecules whose target CpG is methylated. All results are presented on the β-value scale multiplied by 100 to ease interpretability as percent change in DNA methylation. To estimate leukocyte composition as proportions, we used a reference panel of isolated leukocytes with the IDOL projection and Houseman method^48^ to estimate cell-type proportions (CD8 + T cells, CD4 + T cells, NK, B-cells, monocytes, and neutrophils)^49^.

### Genotyping of samples

Genotyping data was measured at 618,540 single nucleotide polymorphism (SNP) sites using the Infinium Global Screening Array (GSA) BeadChips according to the Illumina’s standard protocol (Illumina). GenomeStudio 2.0 Genotyping software was used to transform the raw intensity files into clusters, and subsequently genotype calls by producing cohort-specific clustering files and manifest GSA-24v1-0_C1 (Version 1 A2, Illumina). We applied standard quality control procedures to the array, and SNPs with a MAF ≤ 5% were removed prior to performing PCA using pca (PCAtools). Horn’s analysis was used to determine how many PCs to retain (n = 2) using the paran function (MASS)^50^. Participants were ascribed the rotated PC1 and PC2 loadings to represent and control for genetic ancestry differences in subsequent analyses. After quality control, a total of 465 participants had complete DNA methylation and genetic ancestry data. EWAS of smoking adjusting for genotype PCs was restricted to the 465 participants with high quality DNA methylation and genotyping data.

### Calculation of epigenetic aging biomarkers

Epigenetic age was calculated using five clocks: the Horvath Pan Tissue, Horvath Skin-Blood, Hannum Blood, PhenoAge, and GrimAge clocks. We calculated all epigenetic aging biomarkers utilizing the online Horvath calculator (http://dnamage.genetics.ucla.edu/) with the advanced analysis option. The outcome of interest was the “*AgeAccelerationResidual*” or residuals resulting from a linear regression model where each DNA methylation clock is regressed on chronological age of each participant. We refer to all acceleration measures as Epigenetic Age Acceleration (EAA) for the specific clock and defined the residuals of the DNA methylation estimate of telomere length linearly regressed on chronological age as DNAmTL adjusted for age. A positive EAA indicates that the estimated epigenetic age is higher than the chronological age (increased biological aging) and a negative DNAmTL adjusted for age reflects a shorter telomere length. In addition, we tested Extrinsic EAA (EEAA) and Intrinsic EAA (IEAA) for Hannum’s and Horvath’s clocks, respectively^51^. The EEAA measure is associated with age-related changes in blood cell counts due to immune system aging and is calculated by upweighting the contributions of age-associated blood cell counts (naive cytotoxic T cells, exhausted cytotoxic T cells, and plasmablasts). The IEAA measure is independent of blood cell counts, represents intrinsic cellular aging, and is calculated by adding immune cell counts in addition to chronological age when calculating regression residuals. Analyses of epigenetic clocks included the 489 participants with high quality DNA methylation data.

### Statistical analysis

We described our study sample using means and proportions for the variables analyzed and evaluated accuracy of all epigenetic aging biomarkers via their empirical correlation with chronological age as well as scatterplots. In a linear EWAS model, we compared current and former smokers to non-smokers via *limma*^52^*,* with each individual CpG on the beta value scale while adjusting for sex, chronological age, BMI, education, household assets, the first two principal components from genetic data, and estimated cell-type composition. We report statistically significant results adjusted for multiple comparisons using both a Bonferroni correction of α = 0.05/790,058, or *P* < 6.33 × 10^–8^ and by controlling the False Discovery Rate at 5% (FDR < 0.05). Additionally, we tested for Differentially Methylated Regions (DMRs) using *DMRcate*^53^ for the comparison of current to non-smokers as well as current to former smokers while adjusting for the same covariates as individual linear models. To test for effect modification by region, we fitted a linear model that incorporated interactions between smoking status (current, former, and non-smokers) and residence in the Nicoya Peninsula as binary. We evaluated EWAS model fit by visualizing quantile–quantile plots of the observed vs. expected *P*-values and estimated the genomic inflation factor (λ). We used *missMethyl*^54^ to test for enrichment of differentially methylated sites across KEGG biological pathways. We summarized results using Manhattan and volcano plots of EWAS as well as a circular genomic plot. To test for EAA, we used linear regression to estimate mean differences between smoking groups across acceleration measures of epigenetic aging as the outcome. Namely, we tested the residuals of regressing each epigenetic clock on chronological age against self-reported smoking behavior (current, former and non-smokers). We also tested associations between ever smoking vs. never smoking for comparability with other studies of EAA. Effect modification was evaluated by fitting a multiplicative term between smoking behavior and Nicoya residency in linear regression models of EAA. We report estimates and 95% confidence intervals (95% CI) as well as unadjusted *P*-values for EAA analyses.

## Results

A total of 489 CRELES study participants had complete data and DNA methylation measurements (Table 1); they had a mean (SD) age of 79.4 years (10.8 years), and 90 (18.4%) lived in the high longevity region of the Nicoya Peninsula. Of all participants, 278 were female (57%), 37 (7.6%) reported currently smoking, 171 (35%) were former smokers, and 281 (57.4%) reported never smoking. The majority of participants only had an elementary school education (69%), and 97 participants (20%) had no formal education. To control for population stratification, we adjusted EWAS models for two principal components from genome-wide SNP arrays, limiting these analyses to 465 study participants. These two principal components significantly differed between the regions (*P* < 1 × 10^–7^), with region of residence explaining 28% and 8% of the variance for the first and second genetic principal components, respectively.

**Table 1:** Characteristics of participants from the Costa Rican Study on Longevity and Healthy Aging (CRELES) included in this study, overall and stratified by residents from the Nicoya Peninsula (Nicoyans) or non-residents (non-Nicoyans).

Participant characteristics	Overall (N = 489)	Non-Nicoyans (N = 399)	Nicoyans (N = 90)
**Sex**			
Male	211 (43%)	165 (41%)	46 (51%)
Female	278 (57%)	234 (59%)	44 (49%)
**Level of education**			
None	97 (20%)	78 (20%)	19 (21%)
Elementary (1–6 years)	338 (69%)	270 (68%)	68 (76%)
Secondary (7–11 years)	27 (6%)	27 (7%)	–
Post-Secondary (> = 12 years)	27 (6%)	24 (6%)	3 (3%)
Household assets (0–10)	7.99 (1.76)	8.13 (1.69)	7.36 (1.93)
BMI (kg/m^2)	25.2 (5.5)	25.7 (5.6)	23.4 (4.7)
Age, (years)	79.4 (10.8)	78 (10.4)	85.3 (10.9)
**Smoking status**			
Current	37 (7.6%)	28 (7%)	9 (10%)
Former	171 (35%)	138 (34.6%)	33 (36.7%)
Never	281 (57.4%)	233 (58.4%)	48 (53.3%)
Genetic principal components (PCs)*			
PC1	− 0.55 (32.69)	− 8.51 (27.17)	37.11 (31.23)
PC2	0.11 (22.32)	3.11 (20.51)	− 12.08 (23.06)
Missing	–	15 (3.8%)	9 (10%)
**Estimated leukocyte composition (%)**			
Neutrophils	54.5 (10.6)	54.7 (10.3)	53.9 (12)
NK	8.1 (3.3)	8 (3.2)	8.6 (3.3)
CD4 + -T	14.1 (5.2)	14.1 (5.3)	14.2 (5.1)
CD8 + -T	10.9 (5.0)	10.9 (5)	10.6 (4.9)
Monocytes	8.3 (2.4)	8.2 (2.2)	8.7 (2.9)
B-cells	5.3 (2.5)	5.3 (2.5)	5.4 (2.5)

Statistics presented are n (%) or mean (SD).

*Missing genetic data for 24 study participants.

### Epigenome-wide association analyses (EWAS) of current smoking

**Figure 1:**
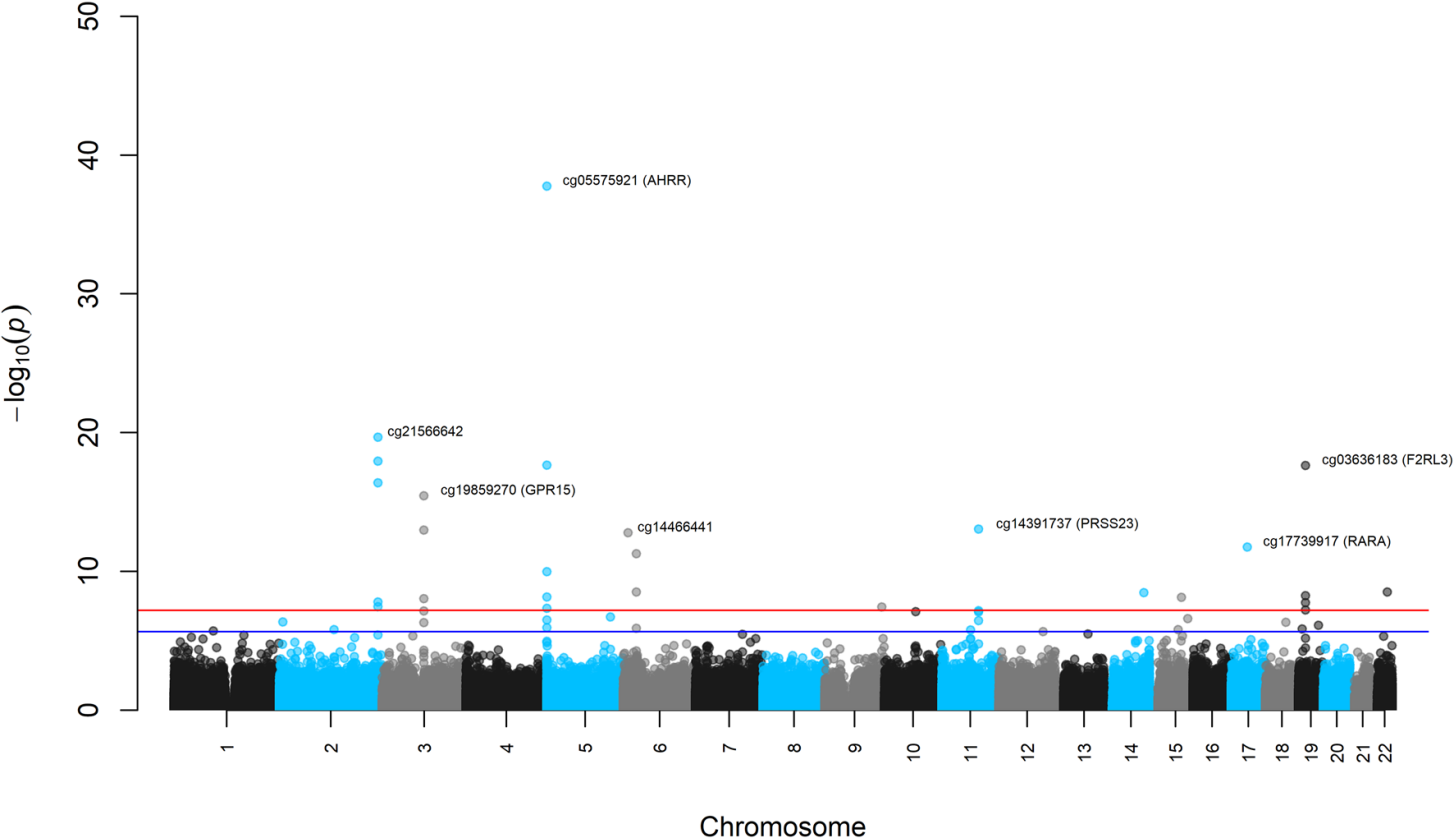
Manhattan plot of epigenome-wide associations among current smokers relative to non-smokers (red line represents a Bonferroni corrected level of significance; blue line represents a false discovery rate of 5%).

**Figure 2:**
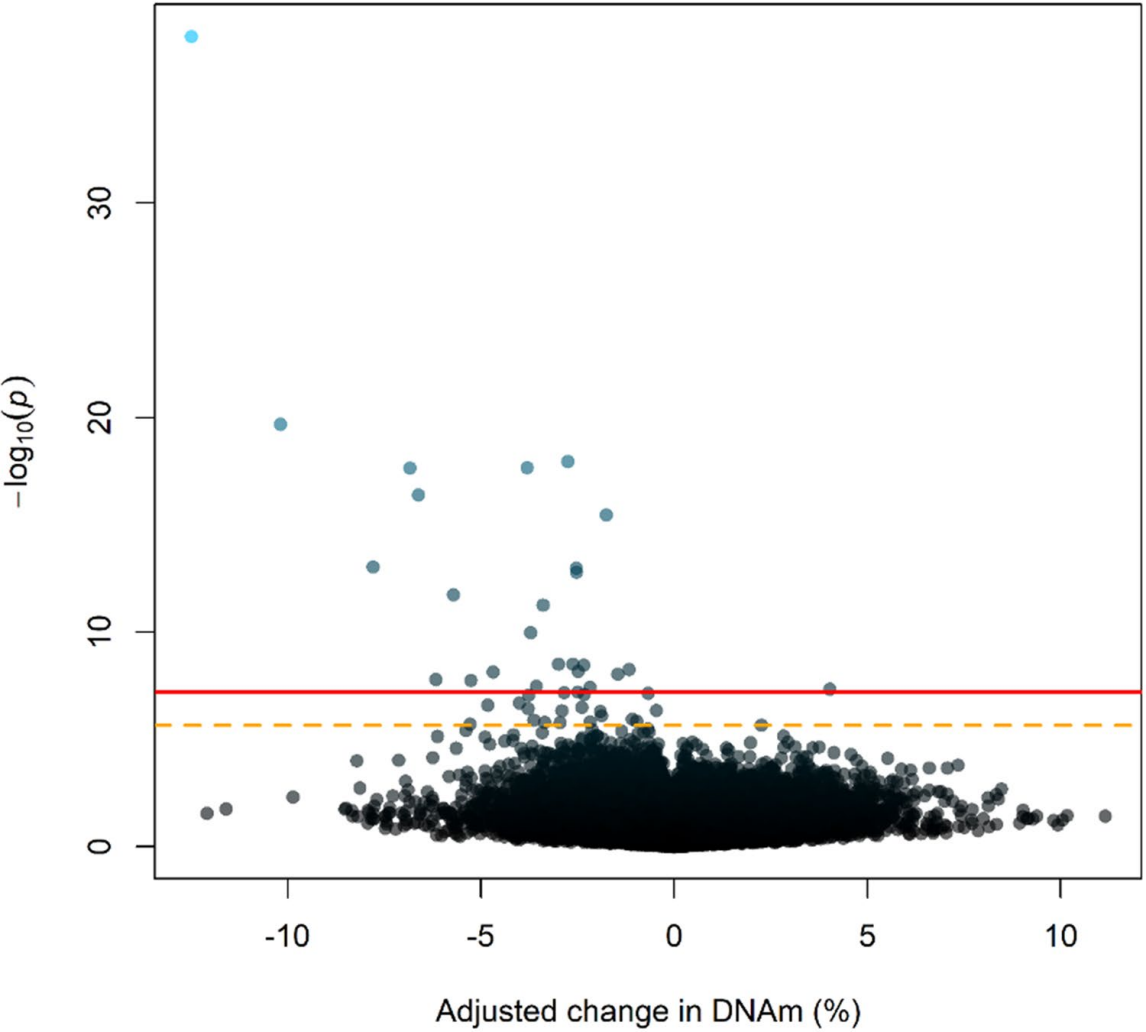
Volcano plot of the -log_10_(p-values) against adjusted regression coefficients for each CpG for the Epigenome-Wide Association Study comparing current smokers to non-smokers (red solid line represents a Bonferroni corrected level of significance; orange dotted line represents a false discovery rate of 5%).

**Figure 3:**
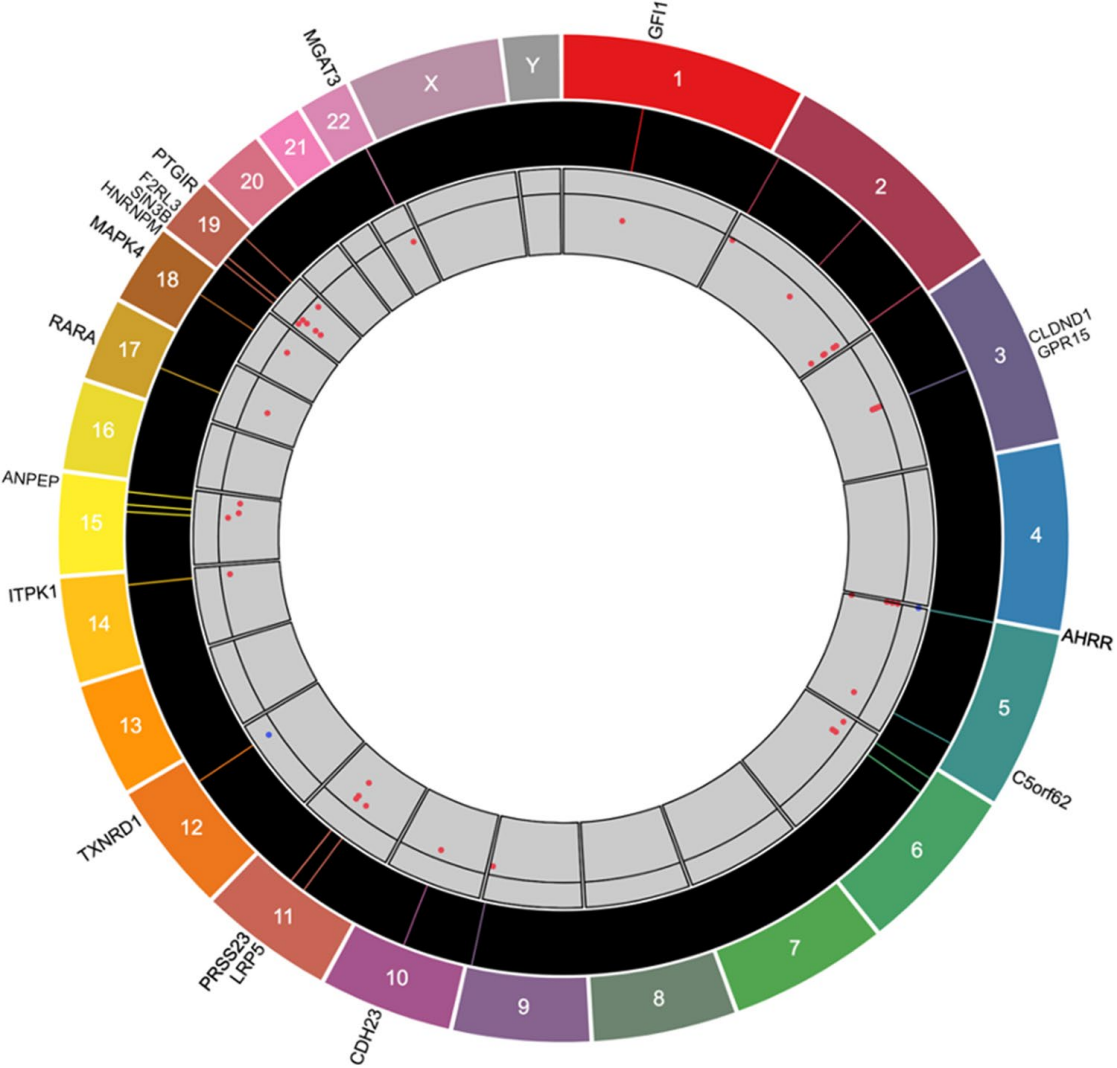
Circular genomics plot for current vs. never smokers. From the outermost ring to the center: gene names, chromosome numbers, locations of CpG sites within each chromosome, and effect sizes of DNA methylation (blue points above the horizontal line of 0 represent hypermethylation; red points below the line represent hypomethylation).

In EWAS adjusted for sex, age, BMI, education, household assets, two principal components of genotypes, and estimated leukocyte composition, a total of 46 CpGs were differentially methylated when comparing current smokers to non-smokers, while adjusting for former smokers. Among the 46 CpGs with a FDR < 0.05, 26 CpGs were statistically significant using a Bonferroni adjusted threshold of *P* < 6.33 × 10^–8^ (α = 0.5/790,058). A Manhattan plot, volcano plot, and circular genomic plot of EWAS results for current vs. non-smokers displaying genomic position, effect sizes and gene annotations are shown in Figs. 1, 2 and 3. Multiple of the top 30 differentially methylated CpGs from this analysis annotated to the same genes (*AHRR, PRSS23, SIN3B, and F2RL3*) and a region in chromosome 2 (chr2: 233,283,010–233,286,229), and almost all were hypomethylated, as shown in Table 2. Among all 46 differentially methylated CpGs found with a FDR < 0.05, 44 (96%) were hypomethylated (Table S1 in Supplementary File 1). The CpGs with the greatest increase (cg07943658; 4.0%) and decrease (cg05575921; − 12.49%) in methylation among current smokers were both annotated to the *AHRR* gene. A comparison of the CpGs found in our study across many others is available in Table S2 in Supplementary File 1. Five CpGs (cg04706667, cg06991517, cg13511253, cg18234441, and cg22812571) have not been previously reported to be differentially methylated in other smoking-related studies, including large meta-analyses (Table S2 in Supplementary File 1). Four of these novel CpGs were hypomethylated and annotated to the mitogen-activated protein kinase 4 (*MAPK4*), the heterogeneous nuclear ribonucleoprotein M (*HNRNPM*), and the prostaglandin I_2_ receptor (*PTGIR*) genes. The remaining CpG was hypermethylated and annotated to the thioredoxin reductase 1 (*TXNRD1*) gene. The directionality of methylation—increased or decreased—for the remaining 41 sites is in alignment with prior findings in the literature, such as the large meta-analysis of Joehanes et al.^55^ and a recent analysis of samples evaluated with the EPIC array from Domingo-Relloso et al. (2020) study^55,56^. The genomic inflation factor (λ = 1.05) and quantile–quantile plot of observed vs. expected *P*-value distribution show no major concerns for the analyses (Supplementary Figure S1).

**Table 2:** Top 30 differentially methylated CpGs for current smokers relative to non-smokers, ranked by smallest *P*-value and sorted by chromosome and position.

CpG	CHR	Position	UCSC Gene Name	Adjusted %-DNA methylation difference^a^	FDRP-value	Relation to Island	Gene region
cg17087741	2	233283010		− 2.75	2.92E−13	N_Shore	
cg03329539	2	233283329		− 3.56	1.15E−03	N_Shore	
cg21566642	2	233284661		− 10.18	8.24E−15	Island	
cg01940273	2	233284934		− 6.61	5.29E−12	Island	
cg22812571	2	233286229		− 6.17	6.05E−04	S_Shore	
cg08064403	3	98240258	*CLDND1*	− 1.46	3.58E−04	N_Shore	Body
cg19859270	3	98251294	*GPR15*	− 1.76	3.89E−11	OpenSea	1st Exon
cg04180924	3	98272064		− 0.67	1.96E−03	OpenSea	
cg02978227	3	98292027		− 2.54	8.86E−09	OpenSea	
cg07943658	5	352001	*AHRR*	4.03	1.43E−03	OpenSea	Body
cg05575921	5	373378	*AHRR*	− 12.49	1.35E−32	N_Shore	Body
cg04551776	5	393366	*AHRR*	− 2.48	2.94E−04	OpenSea	Body
cg25648203	5	395444	*AHRR*	− 3.81	3.54E−13	OpenSea	Body
cg21161138	5	399360	*AHRR*	− 3.71	6.23E−06	OpenSea	Body
cg14466441	6	11392193		− 2.52	1.26E−08	OpenSea	
cg24859433	6	30720203		− 3.39	3.54E−07	OpenSea	
cg15342087	6	30720209		− 2.62	1.63E−04	OpenSea	
cg26768182	9	134272679		− 2.18	1.21E−03	S_Shelf	
cg10750182	10	73497514	*C10orf105*	− 2.33	2.16E−03	OpenSea	5' UTR
cg11660018	11	86510915	*PRSS23*	− 3.76	2.22E−03	N_Shore	TSS1500
cg14391737	11	86513429	*PRSS23*	− 7.79	8.86E−09	S_Shore	5' UTR
cg00475490	11	86517110	*PRSS23*	− 2.85	1.91E−03	OpenSea	5' UTR
cg05284742	14	93552128	*ITPK1*	− 2.34	1.65E−04	OpenSea	Body
cg18110140	15	75350380		− 4.69	2.98E−04	OpenSea	
cg17739917	17	38477572	*RARA*	− 5.71	1.26E−07	S_Shelf	5' UTR
cg03384915	19	16986822	*SIN3B*	− 1.16	2.50E−04	Island	Body
cg14712058	19	16988083	*SIN3B*	− 2.49	1.86E−03	N_Shore	Body
cg21911711	19	16998668	*F2RL3*	− 5.26	6.36E−04	N_Shore	TSS1500
cg03636183	19	17000585	*F2RL3*	− 6.84	3.54E−13	N_Shore	Body
cg05086879	22	39861490	*MGAT3*	− 3	1.63E−04	OpenSea	5'UTR

^a^Linear regression models adjusted for sex, age, BMI, education, household assets, two principal components from genetic data, and estimated cell-type composition (CD8 + -T, CD4 + -T, NK, B-cell, monocytes, and neutrophils).

### EWAS of former smoking

For the adjusted EWAS comparing former to non-smokers while adjusting for current smoking, only one CpG site (cg05575921; *AHRR*) was found to have a significant association with smoking, with a smaller magnitude of effect (− 2.93%). The quantile–quantile *P*-value and Manhattan plots for the EWAS of former smokers relative to non-smokers are shown in Supplementary Figures S2 and S3, respectively.

### DMRs among current smokers and modification of associations by longevity region

Testing for DMRs yielded five DMRs hypomethylated among current smokers relative to non-smokers. Two DMRs annotated to the *AHRR* gene and a single DMR was observed for the *F2RL3,* the Small Nucleolar RNA, H/ACA Box 20 (*SNORA20*), and Small Cajal Body-Specific RNA 6 (*SCARNA6*)/Small nucleolar RNA (SNORD55/SNORD39) genes. Results of DMR analyses are shown in Table 3. No DMRs were found for former smokers relative to non-smokers. Among the differentially methylated CpGs for current smokers relative to non-smokers, three KEGG biological pathways were marginally enriched or overrepresented (*P*_unadjusted_ < 0.05), with more than one gene differentially methylated: *hsa05200* or “Pathways in cancer”, *hsa04611* “Platelet activation,” and *hsa04080* “Neuroactive ligand-receptor interaction.” However, these results did not survive multiple testing adjustments.

**Table 3:** Differentially methylated regions (DMRs) associated with current smoking status relative to non-smokers.

Chromosome	Start	End	Width	CpGs	Stouffer *P*-value	Mean %-DNAm difference at DMR	Overlapping genes
chr2	233283010	233286229	3220	12	3.23 × 10^–13^	− 3.29	*SCARNA6, SNORD39*
chr5	373378	374093	716	3	5.38 × 10^–7^	− 4.34	*AHRR*
chr5	395444	395717	274	2	9.30 × 10^–5^	− 2.08	*AHRR*
chr6	30719807	30720484	678	6	0.017	− 1.64	*SNORA20*
chr19	17000585	17001002	418	3	0.022	− 2.29	*F2RL3*

In EWAS analyses with interactions between smoking (current, former, and never) and longevity region residency, there was no statistical evidence that smoking related DNAm signatures differed between Nicoyan and non-Nicoyan smokers after adjusting for multiple testing (Supplementary Figure S4).

**Figure 4:**
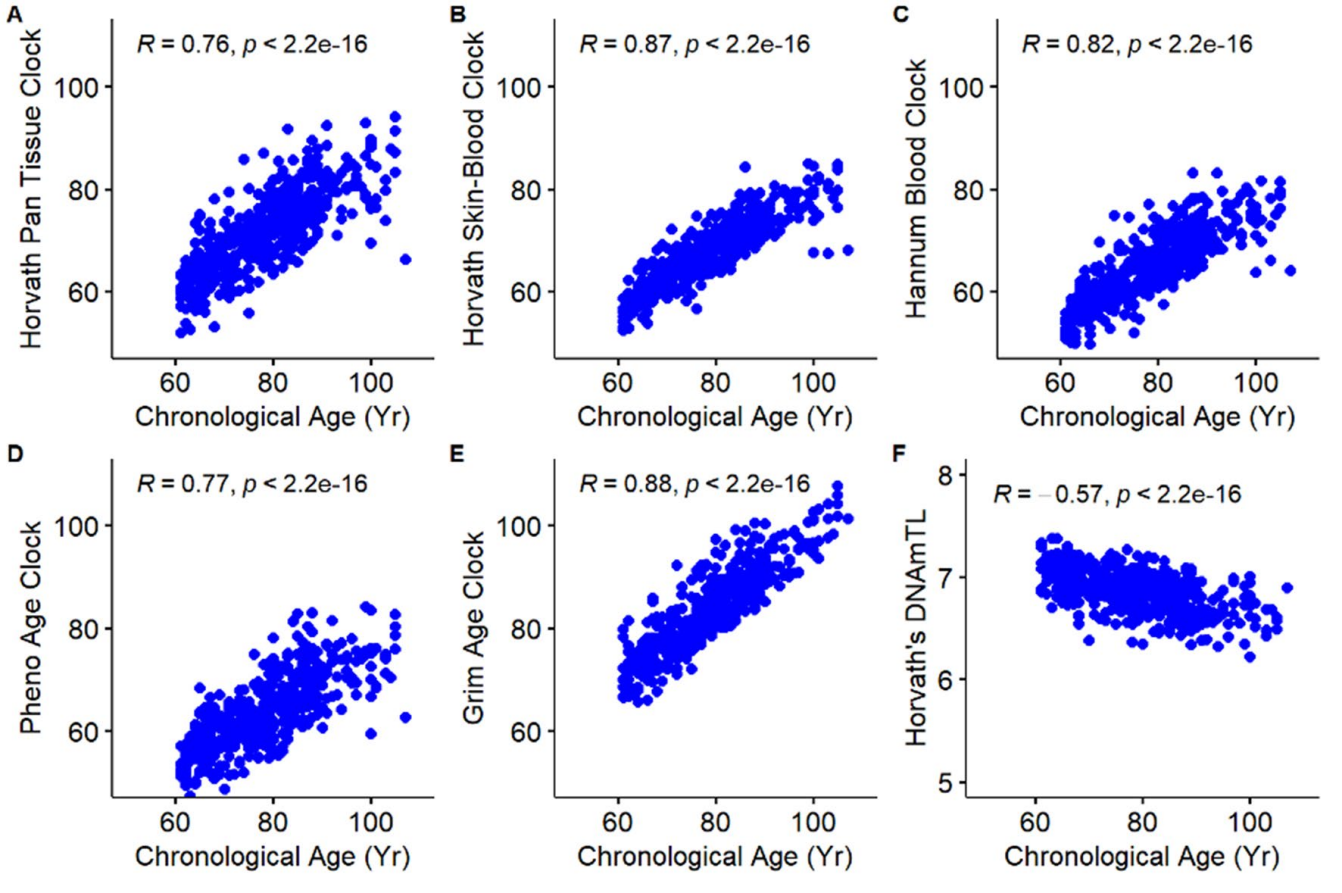
Scatterplots and correlation coefficients of DNA methylation aging biomarkers (epigenetic clocks) and chronological age in years: **(A)** Horvath’s Clock, **(B)** Horvath’s Skin-Blood Clock, **(C)** Hannum’s Clock, **(D)** PhenoAge Clock, **(E)** GrimAge Clock, and **(F)** Horvath’s DNAm telomere length (DNAmTL).

Correlation plots comparing each study participant’s epigenetic age, as determined by five different epigenetic clocks, DNAmTL biomarker, and chronological age are displayed in Fig. 4. Overall, there were strong, positive, and significant correlations between chronological age and epigenetic age for the Horvath Pan Tissue (*r* = 0.76), Skin-Blood (*r* = 0.87), Hannum Blood (*r* = 0.82), PhenoAge (*r* = 0.77), and the GrimAge (*r* = 0.88) clocks. Of note, the GrimAge clock includes chronological age as an input. A moderate negative correlation was observed between DNAmTL estimates and chronological age (*r* = − 0.57). As expected, the EAA measures were uncorrelated with chronological ages.

### Epigenetic age acceleration for ever versus never smokers

**Figure 5:**
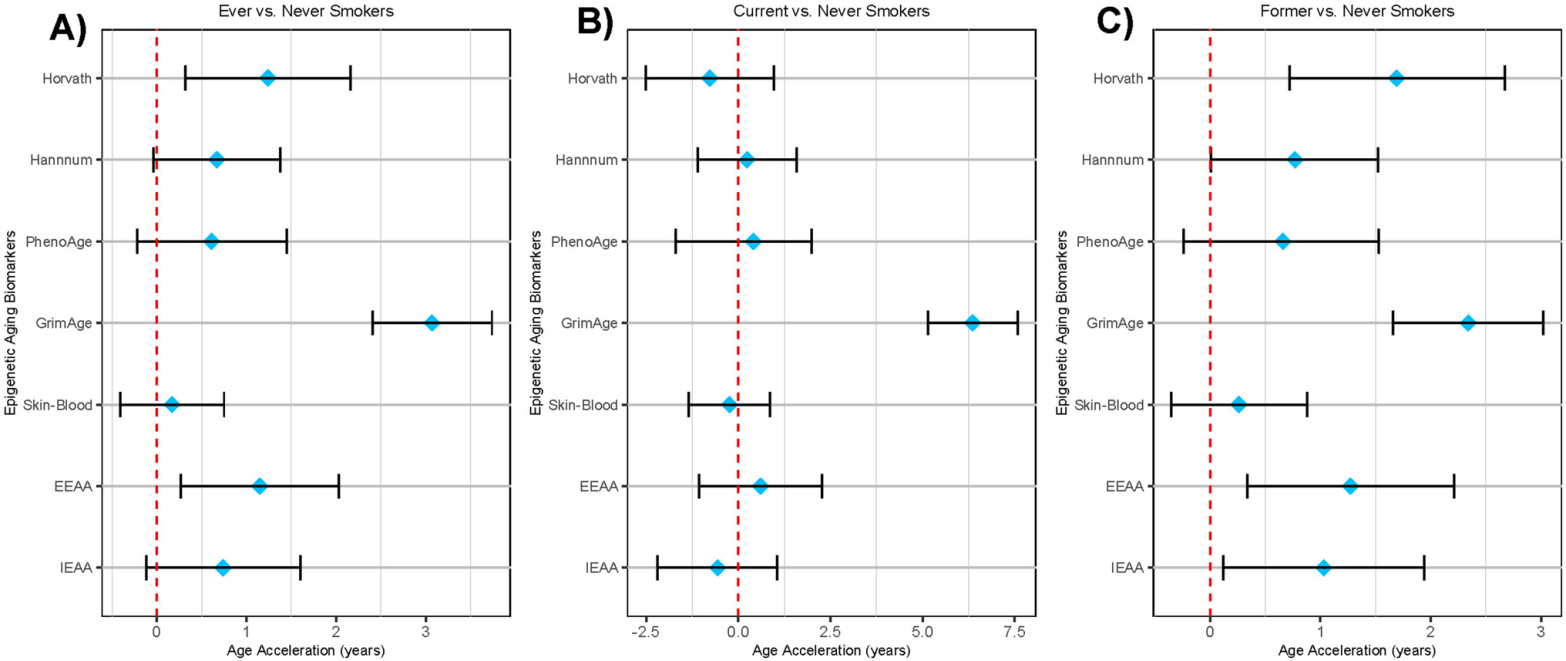
Estimated differences in years of epigenetic age acceleration when comparing: **(A)** ever smokers to never smokers, **(B)** current smokers to never smokers, and **(C)** former smokers to never smokers. Diamonds represent point estimates, error bars represent 95% confidence intervals, and red dashed line represent the null hypothesis. The telomere length biomarker (DNAmTL) was excluded due to scaling.

To compare our data to previous reports, we first tested differences in epigenetic age acceleration when comparing ever smokers, that included current and former smokers, to never-smokers (Fig. 5A); the data are also displayed in Supplementary Table S3. The highest epigenetic age acceleration among ever smokers was found for the GrimAge clock (3.07 years, 95% CI 2.41, 3.74), which incorporates DNA methylation estimated smoking pack-years in its calculation, so this was expected. All clocks showed the same trend of increased age acceleration among smokers, with the Horvath Clock and Extrinsic EAA measure demonstrating significantly increased EAA: 1.24 years (95% CI 0.32, 2.16) and 1.15 years (95% CI 0.27, 2.03), respectively. Additionally, smokers had on average 0.04 kb shorter (95% CI − 0.07, − 0.01) DNA methylation estimates of telomere length residuals after adjusting for chronological age. Similar results were observed when stratifying models with participants from the Nicoya region, with the exception that PhenoAge was significantly accelerated among ever smokers compared to non-smokers from the Nicoya Peninsula (2.11 years; 95% CI 0.14, 4.08) but not in the other region (*P* = 0.44). However, no effect modification of smoking on epigenetic aging was observed for the longevity region in multiplicative interaction models (*P* > 0.05).

### Epigenetic age acceleration for current and former smokers

Furthermore, we tested differences between current and former smokers relative to non-smokers across all age acceleration measures (Fig. 5B,C) and stratified by Nicoya residence (Table S4). Epigenetic age acceleration estimates among current smokers relative to non-smokers were not statistically significant, inconsistent in directionality, and relatively weak in strength, except for GrimAge, which was expected due to the incorporation of pack-years in its calculation. For former smokers, acceleration measures for the Horvath Pan Tissue, Hannum Blood, EEAA, and IEAA measures were positive and statistically significant: 1.69 years (95% CI 0.72, 2.67), 0.77 years (95% CI 0.01, 1.52), 1.27 years (95% CI 0.34, 2.21), and 1.03 (95% CI 0.12, 1.94), respectively. Overall, the greatest age acceleration was again observed for the GrimAge clock: 6.36 years (95% CI 5.14, 7.58) for current smokers and 2.34 years (95% CI 1.66, 3.02) for former smokers. After regressing age out from DNAmTL, a 0.04 kb lower (95% CI − 0.08, − 0.01) estimate was observed among former smokers compared to current smokers. No significant effect modification was observed for the relationship between smoking and epigenetic aging markers by longevity region residency (*P* > 0.05).

Overall, stratified results were consistent between Nicoyans and non-Nicoyans despite the sample size of the former group. However, the EAA for the PhenoAge clock was significantly accelerated, 2.28 years (95% CI 0.11, 4.44) among former smokers in Nicoya compared to 0.39 years (95% CI − 0.58, 1.36) for former smokers not from Nicoya. Some prior studies have found negative and/or insignificant differences in age acceleration when using whole blood samples from former, current, and never smokers, as summarized in Supplementary Table S5.

## Discussion

In this study of a Latino adult population living in Costa Rica, including residents from the high longevity region of the Nicoya Peninsula, we investigated associations between current, former, and never smoking status with DNA methylation signatures and epigenetic age acceleration. Our findings replicated previously reported associations within the *AHRR, PRSS23*, *SIN3B*, and *F2RL3* genes and found 5 novel signatures, which annotated to the *MAPK4*, *HNRNPM*, *PTGIR*, and *TXNRD1* genes. Lastly, our results provided strong support that former smokers have accelerated epigenetic aging for Horvath’s and Hannum’s epigenetic clocks as well as extrinsic and intrinsic measures of aging. Consistently, former smokers had shorter DNA methylation estimates of telomere length adjusted for age. In addition, we did not observe significant epigenetic age acceleration among current smokers, except for GrimAge, which could be due to small sample size and suggest importance of differentiating current and past smoking habits to test associations.

In the EWAS comparing current smokers to non-smokers, we found 41 CpG sites that were replicated in previous studies as well as five novel CpGs. The directionality of methylation for the overlapping CpG sites aligns with findings from previous studies^55–61^. The majority of the significant CpG sites in this study overlapped with findings from a study on cigarette smoking among American Indian adults^56^, and fewer overlapped with findings from a study of African American women^58^, both using the EPIC array. The five novel sites that we found have not been reported in other studies, even those that similarly used the 850K EPIC Illumina BeadChip and included participants from a racial minority population^56,58,62^. Four of these sites were hypomethylated and annotated to the *MAPK4*, *HNRNPM*, and *PTGIR* genes. MAPK4 is an atypical kinase involved with the AKT/mTOR signaling pathway, and overexpression is associated with acute lung injury and cancers^63,64^. Similarly, the HNRNPM and PTGIR proteins have been shown to promote cancerous cell growth and be associated with poorer oncogenic outcomes^65,66^. The latter is also involved with vascular remodeling, and its loss of function may increase risk for vessel stenosis and dissection^67^. If *MAPK4* (TSS1500), *HNRNPM*, and *PTGIR* hypomethylation among smokers—what we observed—leads to greater protein expression, this could partially explain their increased risk for lung and heart diseases and several cancers. The remaining novel CpG site was hypermethylated and annotated to the *TXNRD1* gene. The TXNRD1 protein is involved in protecting cells from reactive oxygen species and also promotes tumor growth and DNA replication^68^. Further study of changes to gene expression arising from epigenetic modifications among smokers can elucidate how environmental tobacco exposure leads to the development of diseases.

Our analysis of epigenetic age acceleration demonstrated that for several biological aging biomarkers, ever smokers experience accelerated aging compared to non-smokers. The varied age acceleration results may be because some clocks are better at capturing adverse health impacts from specific environmental stimuli than other clocks. For example, the largest age acceleration associated with smoking was found for the GrimAge clock, which is in alignment with previous studies demonstrating the clock’s success at predicting mortality associated with smoking exposure^32,69^. We expected this result, as pack-years is used in the estimation of GrimAge years^32^. In all other clocks, age acceleration was positive and statistically significant for former smokers but not current smokers. This might be explained by delayed effects of smoking on the development of negative health outcomes or active compensation in the epigenome of current smokers for the toxic exposure of cigarette smoke, which could explain the mostly null age accelerations. Alternately, current smokers that made it into the study at the older ages of recruitment might be uniquely unaffected by smoking. For example, former smokers might have ceased to smoke due to declining health while current smokers continued as they were unaffected. This might be partially supported by genetic findings showing that long-lived smokers carry variants that may confer protection^70^. This hypothesis warrants further testing in the context of epigenetic clocks. Interestingly, the opposite was true in site-by-site analyses for EWAS, where we observed strong associations among current smokers compared to former smokers.

Overall, there are inconsistent results across existing studies that have used a variety of epigenetic clocks on whole blood samples to assess the effects of smoking status on epigenetic age acceleration. Many studies that found null age acceleration results comparing smokers to non-smokers did not stratify smokers into current and former smokers^25,71,72^. This may lead to results that misrepresent the epigenetic effects of cigarette smoke exposure. Also, inconsistencies in those studies and this one may be due to participants’ duration and intensity of smoking as well as time since smoking cessation, which are individual-level factors that can affect epigenetic age acceleration outcomes^73–75^. Among ever smokers, some studies found positive results that are not statistically significant or slightly negative epigenetic age acceleration^25,71,73,76,77^. We also observed non-significant epigenetic age deacceleration among current smokers for Horvath’s Clock, the Skin-Blood Clock, and the IEAA measure. In contrast to our study, previous analyses of smoking have found no associations with IEAA^25,28^. Our analysis builds on previous evidence by including a comprehensive set of epigenetic age acceleration outcomes to assess the consistency of results across different epigenetic clocks. While we did not find evidence of effect modification by the high longevity region, future research should evaluate associations between epigenetic aging and plant-based diets, consistent physical activity, and sociocultural connectedness—factors found to be increased in Blue Zones^78^. For example, residents from Nicoya report higher levels of physical activity and greater intake of fruits and vegetables, black beans, corn tortillas and rice^79^.

This study has some limitations. Due to the study design, we were unable to determine temporality and results might be influenced by recall bias. Also, the associations that we found could be explained by a common factor that was unaccounted for, as residual confounding is a common source of bias in observational studies. However, given the replication of previous smoking signatures, we think this is less likely. To mitigate the chance that this may occur, we controlled for sociodemographic characteristics, genetic principal components, and estimated cell-type composition in EWAS models. Another limitation is that only 90 of the study participants lived in the Nicoya region and only 9 were current smokers, which reduces the statistical power of the study to detect differences in associations among smokers and non-smokers living inside and outside the region. The sample from Nicoya was also older, which might introduce survivor bias regarding the smokers included in this study. Importantly, cigarette smoking data was self-reported by participants, which may introduce bias in the exposure assessment, but we expect this to be non-differential relative to DNA methylation or epigenetic aging measures.

In this EWAS of smoking conducted with a Latino cohort, we found five novel differentially methylated CpG sites among smokers. It also replicated several DNA methylation signatures of current smoking found in previous studies, such as hypomethylation of CpG sites annotated to the *AHRR*, *F2RL3*, *SIN3B*, and *PRSS23* genes. In our study, former smokers exhibited consistent increased epigenetic age acceleration for several epigenetic clocks. Future studies with diverse populations and transcriptome analysis would assist in determining how environmental factors increase health risks among smokers and affect health disparities. Importantly, addressing factors that might promote resilient epigenomes even in the presence of harmful exposure can help optimize public health interventions.

## Supplementary Information

Supplementary Information 1.

Supplementary Information 2.

## Data Availability

Public-use version of the CRELES data is available from the Inter-University Consortium for Political and Social Research (ICPSR) repository (http://doi.org/10.3886/ICPSR31263.v1). Since data DNA methylation and the complementary sample of centenarians in Nicoya are not currently part of the public-use, requests for restricted access to data can be submitted at http://www.creles.berkeley.edu/ following institutional review approval.
