# Supplementary material for: Epigenome-wide association study and epigenetic age acceleration associated with cigarette smoking among Costa Rican adults
Source: Sci Rep. 2022 Mar 11;12:4277. doi: 10.1038/s41598-022-08160-w (PMC8917214; doi:10.1038/s41598-022-08160-w)
Supplement: Supplementary file 2 — Supplementary Information 2. [file 41598_2022_8160_MOESM2_ESM.docx]

**Supplementary Material**

**Figure S1.** Quantile-quantile plot of observed and expected distribution of p-values and genomic inflation factor (λ) for the Epigenome-Wide Association Study (EWAS) of current smokers relative to non-smokers from fully adjusted models.


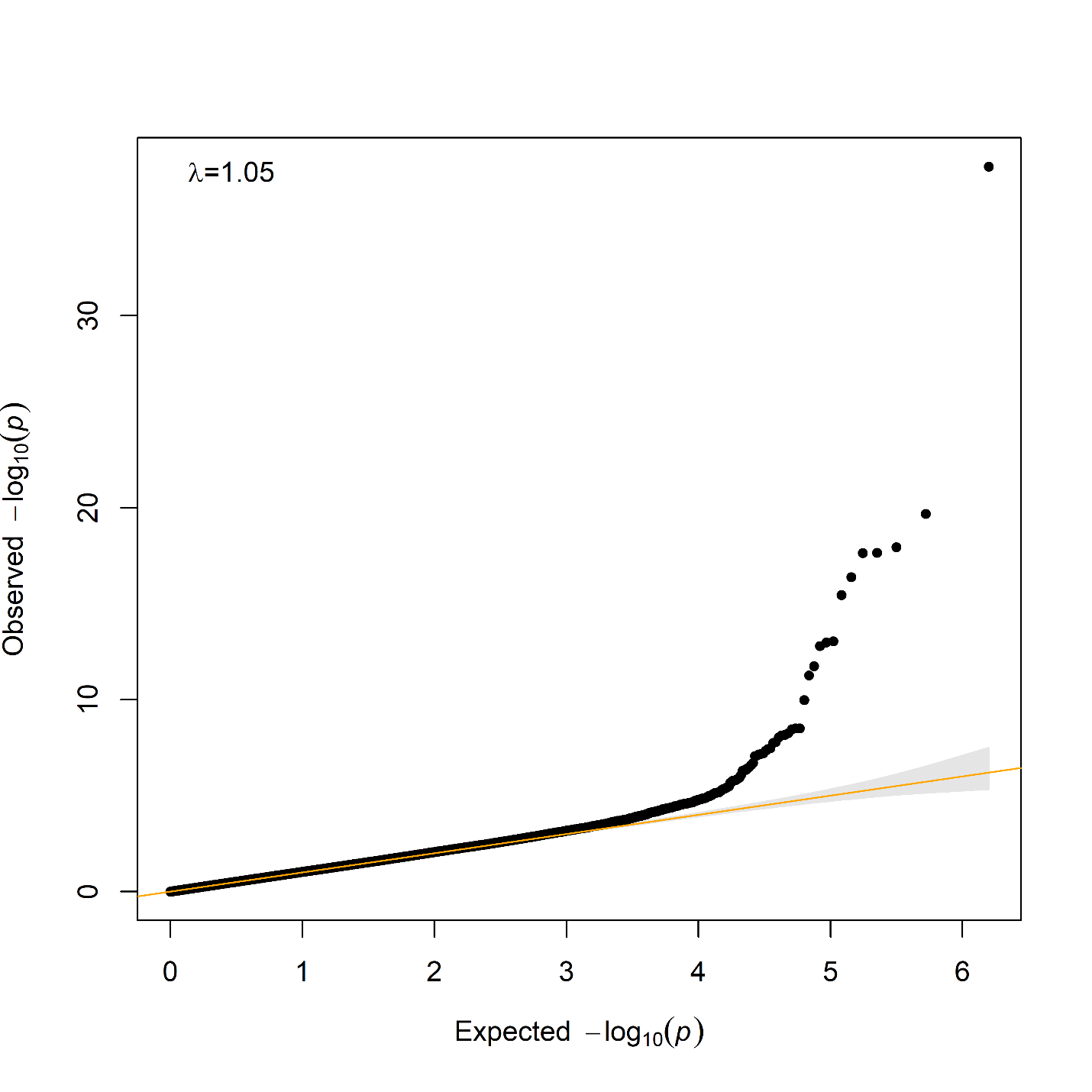


**Figure S2.** Quantile-quantile plot of observed and expected distribution of p-values and genomic inflation factor (λ) for the Epigenome-Wide Association Study (EWAS) of former smokers relative to non-smokers.


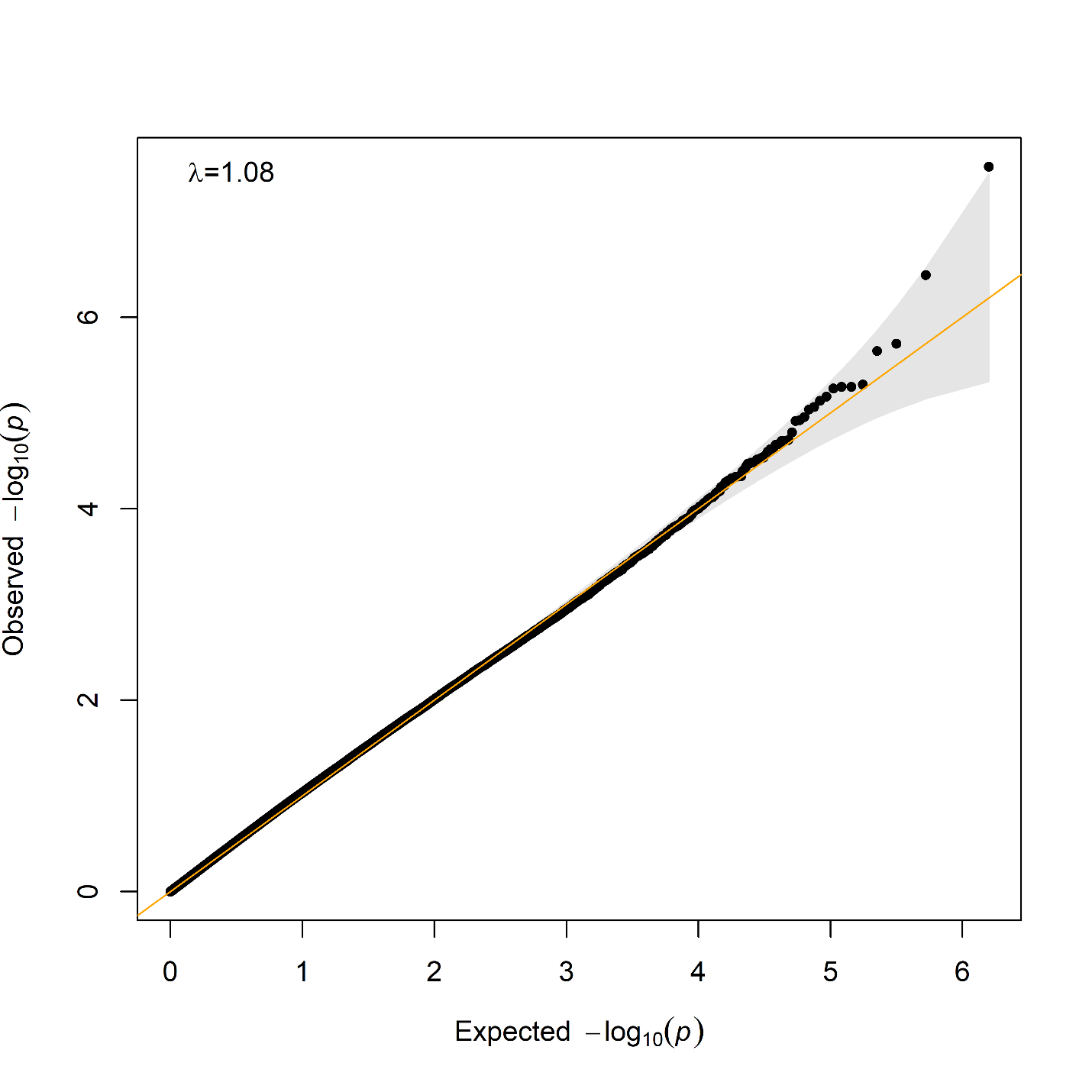


**Figure S3.** Manhattan plot of the Epigenome-Wide Association Study (EWAS) of former smokers relative to non-smokers (red line represents a Bonferroni corrected level of significance).


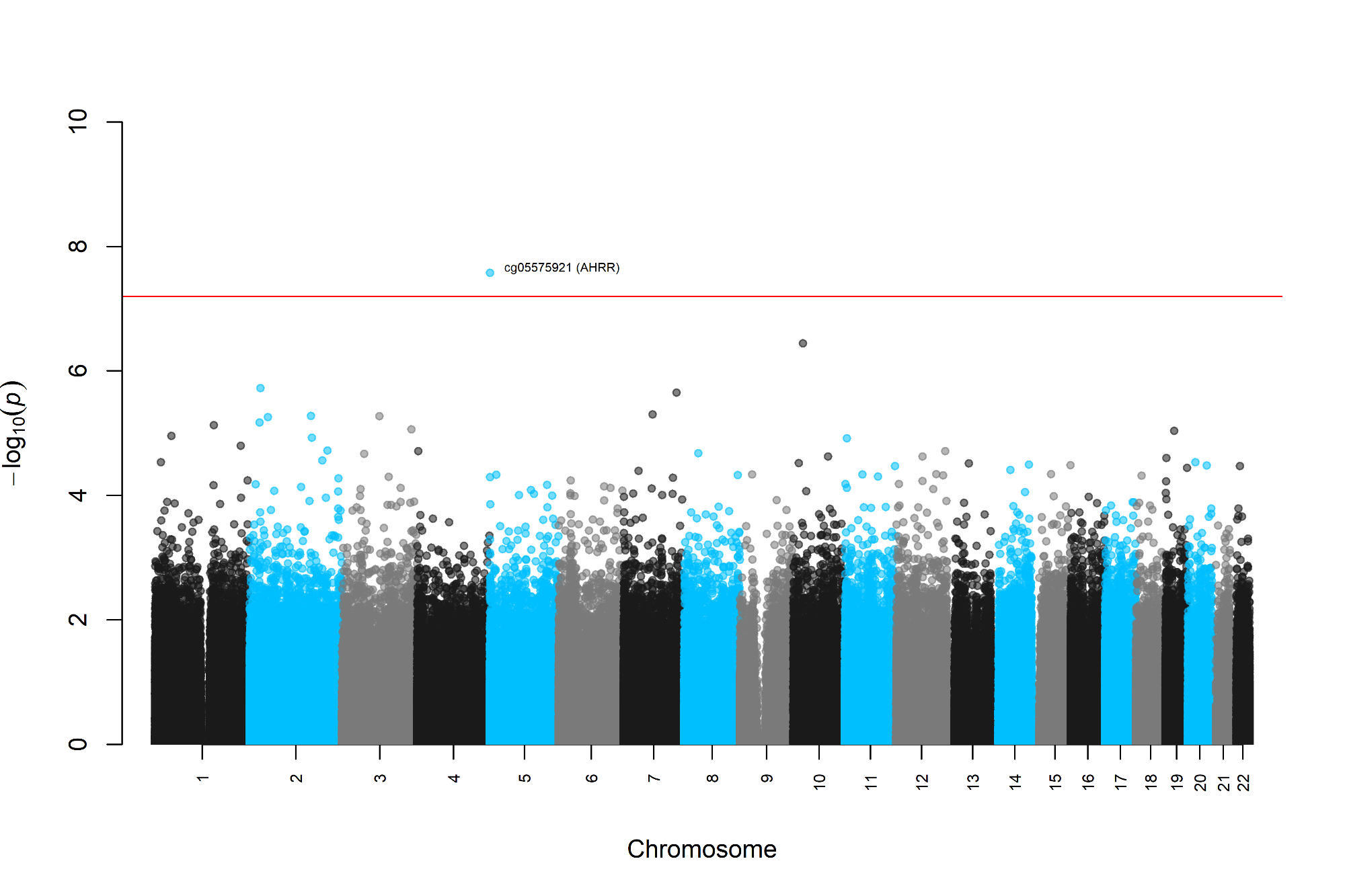


**Figure S4.** Quantile-quantile plot of observed and expected distribution of p-values and genomic inflation factor (λ) for the EWAS with the statistical interaction between current smoking and Blue Zone residence.


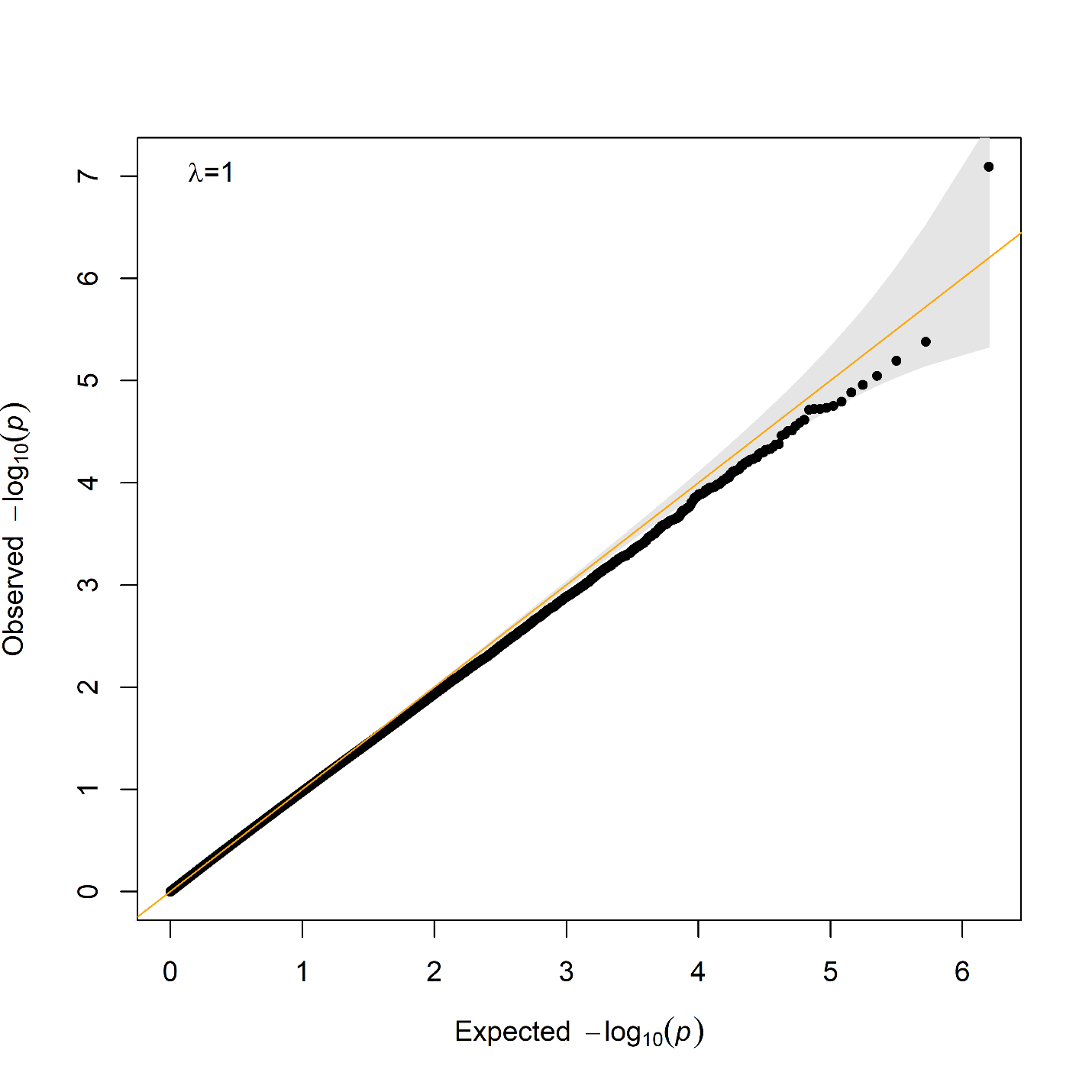


**Table S3.** Estimated differences in years of Epigenetic Age Acceleration (EAA) comparing ever smokers (>100 cigarettes in their lifetime) to never smokers, overall and stratified by region.

|  | **Overall**  **(N=489)** | | | **Non-Nicoyan**  **(N=399)** | | | **Nicoyan**  **(N=90)** | | |
| --- | --- | --- | --- | --- | --- | --- | --- | --- | --- |
| **Epigenetic Aging Outcome** | **Ever smoked *vs.* never** | **95% CI** | ***P*** | **Ever smoked *vs.* never** | **95% CI** | **P-value** | **Ever smoked *vs.* never** | **95% CI** | ***P*** |
| Horvath Pan Tissue | **1.24*** | **(0.32, 2.16)** | **0.008** | **1.21*** | **(0.23, 2.20)** | **0.016** | 1.46 | (-1.20, 4.11) | 0.28 |
| Hannum Blood | 0.67 | (-0.04, 1.38) | 0.06 | 0.59 | (-0.18, 1.35) | 0.13 | 1.15 | (-0.81, 3.11) | 0.24 |
| PhenoAge | 0.61 | (-0.22, 1.45) | 0.15 | 0.36 | (-0.56, 1.28) | 0.44 | **2.11*** | **(0.14, 4.08)** | **0.036** |
| GrimAge | **3.07*** | **(2.41**, **3.74)** | **<2x10^-16^** | **2.94*** | **(2.21, 3.67)** | **2.97x10^-14^** | **3.74*** | **(2.14, 5.34)** | **1.55x10^-5^** |
| Skin-Blood | 0.17 | (-0.41, 0.75) | 0.56 | 0.23 | (-0.41, 0.88) | 0.48 | -0.16 | (-1.46, 1.15) | 0.81 |
| EEAA | **1.15*** | **(0.27, 2.03)** | **0.01** | **0.99*** | **(0.05, 1.9)** | **0.04** | 2.07 | (-0.37, 4.51) | 0.09 |
| IEAA | 0.74 | (-0.12, 1.60) | 0.09 | 0.74 | (-0.19, 1.67) | 0.12 | 0.77 | (-1.60, 3.13) | 0.52 |
| DNAmTL adjusted for age | **-0.04*** | **(-0.07, -0.01)** | **0.0052** | **-0.05*** | **(-0.08, -0.01)** | **0.006** | -0.04 | (-0.13, 0.04) | 0.33 |

***Bold denotes statistically significant associations (*P*<0.05)**

**Table S4.** Estimated differences in years of epigenetic age acceleration (EAA) among current and former smokers compared to non-smokers, overall and stratified by region.

|  | **Overall (N=489)** | | | **Non-Nicoyan (N=399)** | | | **Nicoyan (N=90)** | | |
| --- | --- | --- | --- | --- | --- | --- | --- | --- | --- |
| **Epigenetic Age Acceleration** | **Smoking Coef.** | **95% CI** | ***P*** | **Smoking Coef.** | **95% CI** | ***P*** | **Smoking Coef.** | **95% CI** | ***P*** |
| Horvath Pan Tissue |  |  |  |  |  |  |  |  |  |
| Current | -0.77 | (-2.51, 0.96) | 0.38 | 0.03 | (-1.90, 1.97) | 0.97 | -3.41 | (-7.43, 0.62) | 0.10 |
| Former | **1.69*** | **(0.72, 2.67)** | **6.72x10^-4^** | **1.45*** | **(0.48, 2.56)** | **0.0063** | **3.14*** | **(0.42, 5.88)** | **0.024** |
| Hannum Blood |  |  |  |  |  |  |  |  |  |
| Current | 0.24 | (-1.10,1.59) | 0.72 | 0.73 | (-0.78, 2.23) | 0.34 | -1.39 | (-4.47, 1.67) | 0.37 |
| Former | **0.77*** | **(0.01, 1.52)** | **0.046** | 0.56 | (-0.25, 1.37) | 0.18 | 2.04 | (-0.05, 4.12) | 0.06 |
| PhenoAge |  |  |  |  |  |  |  |  |  |
| Current | 0.41 | (-1.17, 1.99) | 0.61 | 0.19 | (-1.62, 2.00) | 0.84 | 1.63 | (-1.79, 4.95) | 0.31 |
| Former | 0.66 | (-0.24, 1.53) | 0.15 | 0.39 | (-0.58,1.36) | 0.43 | **2.28*** | **(0.11, 4.44)** | **0.039** |
| GrimAge |  |  |  |  |  |  |  |  |  |
| Current | **6.36*** | **(5.14, 7.58)** | **<2x10^-16^** | **6.61*** | **(5.22, 7.99)** | **<2x10^-16^** | **5.33** | **(2.79, 7.89)** | **8.59x10^-5^** |
| Former | **2.34*** | **(1.66, 3.02)** | **4.69x10^-11^** | **2.20*** | **(1.45, 2.94)** | **1.23x10^-8^** | **3.18*** | **(1.45, 4.91)** | **4.77x10^-4^** |
| Skin-Blood |  |  |  |  |  |  |  |  |  |
| Current | -0.24 | (-1.34, 0.86) | 0.67 | 0.11 | (-1.16, 1.39) | 0.86 | -1.44 | (-3.52, 0.63) | 0.17 |
| Former | 0.26 | (-0.35, 0.88) | 0.40 | 0.25 | (-0.42, 0.94) | 0.46 | 0.29 | (-1.30, 1.51) | 0.68 |
| EEAA |  |  |  |  |  |  |  |  |  |
| Current | 0.60 | (-1.06, 2.27) | 0.48 | 0.96 | (-0.91, 2.82) | 0.31 | -0.39 | (-4.27, 3.47) | 0.84 |
| Former | **1.27*** | **(0.34, 2.21)** | **0.0077** | 1.00 | (-0.003, 2.00) | 0.051 | **2.93*** | **(0.30, 5.56)** | **0.029** |
| IEAA |  |  |  |  |  |  |  |  |  |
| Current | -0.56 | (-2.19, 1.06) | 0.50 | 0.15 | (-1.67, 1.98) | 0.87 | -2.97 | (-6.61, 0.67) | 0.11 |
| Former | **1.03*** | **(0.12, 1.94)** | **0.026** | 0.86 | (-0.12, 1.84) | 0.09 | 2.07 | (-0.41, 4.54) | 0.10 |
| DNAmTL adjusted for age |  |  |  |  |  |  |  |  |  |
| Current | -0.05 | (-0.11, 0.01) | 0.15 | -0.05 | (-0.12, 0.01) | 0.10 | -0.03 | (-0.17, 0.11) | 0.69 |
| Former | **-0.04*** | **(-0.08, -0.01)** | **0.0082** | **-0.04*** | **(-0.08, -0.01)** | **0.013** | -0.05 | (-0.14, 0.05) | 0.33 |

***Bold denotes statistically significant associations (*P*<0.05)**

**Table S5.** Summary of findings from studies on cigarette smoking and epigenetic age acceleration.

| **Study** | **Tissue (Sample Size)** | **Smoking Assessment** | **Clock(s)** | **Results (years)** |
| --- | --- | --- | --- | --- |
| Gao (2016) | Whole Blood (N=978) | Current and Former compared to Never; Cumulative smoking | 1. Horvath  2. Hannum | 1. Current (-0.13), Former (0.10), and Cumulative (0.0020)  2. Current (-0.34), Former (-0.51), and Cumulative (0.0099) |
| Yang (2019) | Whole Blood (N=1,214) | Current and Former compared to Never; Cumulative smoking | PhenoAge | Current (2.69), Former (0.17), and **Cumulative (0.04*)** |
| Quach (2017) | Whole Blood (N=4,142) | Current compared to Never | 1. EEAA (Hannum)  2. IEAA (Horvath) | 1. Current (0.30)  2. Current (0.26) |
| Horvath (2016) | Whole Blood (N=1,462) | Former and Never compared to Current | 1. EEAA (Hannum)  2. IEAA (Horvath) | 1. Former (-0.104) and Never (-0.122)  2. Former (-0.573) and Never (-0.376) |
| Simons (2016) | Whole Blood (N=100) | Current (within last year) vs Never | Hannum | Current (-0.08) |
| Simpkin (2017) | 1. Whole Blood (N=152)  2. Buccal Cells (N=790) | Never and Former compared to Current | Horvath | 1. **Never (1.86*)** and Former **(1.88*)**  2. Never (-0.16) and Former (+ 0.83) |
| Dugué (2018) | Whole Blood (N=2,818) | Former smoker who had quit ≤15 years prior, former smoker who had quit >15 years prior, current smoker of ≤20 cigarettes/day, and current smoker of >20 cigarettes/day compared to Never | 1. EEAA (Hannum)  2. IEAA (Horvath) | 1. **Former ≤15 years (0.65*)**, Former >15 years (-0.03), **Current ≤20 cigarettes/day (2.12*)**, **Current >20 cigarettes/day (1.26*)**  2. **Former ≤15 years (0.78*)**, Former >15 years (0.45), **Current ≤20 cigarettes/day (1.33*)**, Current >20 cigarettes/day (0.86) |
| McCartney (2018) | Whole Blood (N=4,997) | Smoking (pack years) | 1. EEAA (Hannum)  2. IEAA (Horvath) | 1. **Pack years (0.059*)**  2. Pack years (0.031) |
| Luo (2020) | Whole Blood (N=331) | Smokers vs. Non-smokers | PhenoAge | **Smoker (1.86*)** |

***Statistically significant in the study**
